# Quantification of α-Thujone and Its Metabolites in Human Urine after Consumption of a Sage Infusion Using Stable Isotope Dilution Assays

**DOI:** 10.3390/toxins10120511

**Published:** 2018-12-03

**Authors:** Irene Thamm, Konrad Tiefenbacher, Michael Rychlik

**Affiliations:** 1 Technical University of Munich, Chair of Analytical Food Chemistry, Maximus-von-Imhof-Forum 2, 85354 Freising, Germany; thamm.irene@googlemail.com; 2 Department of Chemistry, University of Basel BPR 1096, Postfach 3350 Mattenstrasse 24a, CH-4002 Basel, Switzerland; konrad.tiefenbacher@unibas.ch; 3 Department of Biosystems Science and Engineering, ETH Zürich, Mattenstrasse 26, CH-4058 Basel, Switzerland; 4 Centre for Nutrition and Food Sciences, Queensland Alliance for Agriculture and Food Innovation, the University of Queensland, Coopers Plains, QLD 4108, Australia

**Keywords:** thujone, 4-hydroxythujone, 7-hydroxythujone, hydroxythujone conjugates, human study

## Abstract

The metabolism of the monoterpene α-thujone was investigated in humans after consumption of sage tea, by analyses of its metabolites 2-hydroxythujone, 4-hydroxythujone, and 7-hydroxythujone in urine. For the quantitation of α-thujone and its metabolites, stable isotope dilution assays were developed. Using *d*_6_-α-thujone as internal standard, we quantified α-thujone by solid phase microextraction GC-MS and the hydroxythujones with *d*_6_-2-hydroxythujone, *d*_6_-4-hydroxythujone, and *d*_6_-7-hydroxythujone after glucuronide/sulfate deconjugation by LC-MS/MS in urine. After the consumption of 575.0 µg α-thujone, the 4-hydroxythujone and 7-hydroxythujone were detectable in the urine of the volunteers under study, after liberation from their glucuronides/sulfates. The 2-Hydroxythujone was not present in any of the volunteer samples above its detection limit. α-Thujone was detectable in a low amount, with a maximum concentration of 94 ng/L for the volunteer with the highest dose of 14.3 µg/kg bw.

## 1. Introduction

Several herbs like sage, thyme, and rosemary contain the monoterpene thujone. However, thujone became famous for being produced by wormwood and, therefore, being a component of the spirit absinthe. The drink was popular in the 19th century, and was prohibited due to concerns about its toxicity. Today it is permitted again in the European Union, with an imposed maximum limit for thujone. The symptoms of absinthism, like hallucinations, depression, and convulsions were connected with thujone, but more recent studies propose that most of these symptoms were caused by alcohol intoxication [1]. Nevertheless, recent studies showed that thujone is neurotoxic and inhibits the gamma-aminobutyric acid A (GABAA) receptor, which leads to convulsions and excitations at higher concentrations in animal studies [2]. 

Until now, there have only been a few studies on the metabolism of α-thujone as the more active isomer. In one of the few studies on this subject, Höld et al. performed in vitro and in vivo studies on mice and rats. They reported the quick metabolism of α-thujone in vitro by mouse liver microsomes, with 7-hydroxythujone (7-OH) as the major metabolite [2]. However, the in vivo studies in rats pointed to 2-hydroxythujone (2-OH) and 4-hydroxythujone (4-OH) as the main metabolites [3]. A study on human hepatic preparations in vitro by Abass et al. identified the action of cytochrome P450 dependent monooxygenases, and demonstrated that human liver microsomes produce 7-OH and 4-OH as major metabolites. All of these studies reported hydroxythujones as important metabolites. However, in vivo evidence of α-thujone metabolism in humans is still missing.

**Figure 1:**
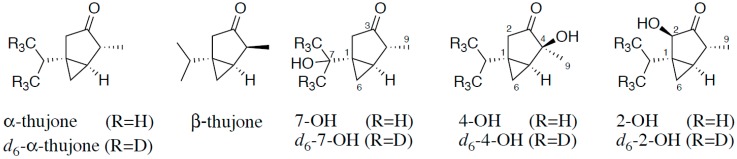
Structure of α-thujone and β-thujone and the hydroxylated metabolites of α-thujone 7-hydroxythujone (7-OH), 4-hydroxythujone (4-OH), and 2-hydroxythujone (2-OH), along with positions of the isotopical labeling of the internal standards.

Therefore, the aim of the present study was to characterize the metabolism of α-thujone in humans in vivo by stable isotope dilution assays (SIDAs). For this purpose we used stable isotopologues of α-thujone and the hydroxythujones 2-OH, 4-OH, and 7-OH as internal standards (IS) (Figure 1), the synthesis of which we reported recently [4]. The development of a SIDA enabled us to detect trace amounts of α-thujone and these metabolites in human urine after consumption of a sage infusion.

## 2. Results

### 2.1. Development of SIDAs for α-Thujone by SPME-GC-MS

For detection of α-thujone in urine, LC-MS/MS was not suitable due to low ionization efficiency. In preliminary experiments we also tested solvent-assisted flavor evaporation prior to GC-MS, and different fibers for solid phase micro extraction (SPME). High sensitivity for SPME-GC-MS was observed when using the Car-PDMS 75 µm fiber. For calibration, analyte/standard mixtures were analyzed to convert area ratios A_A_/A_IS_ of analyte and IS to mass ratio m_A_/m_IS_. Thus, we obtained two response equations for different concentration areas: (I) α-thujone < 0.3 ng/L, m_A_/m_IS_ = 0.7352, A_A_/A_IS_—0.0014; and (II) α-thujone 0.3–4.2 ng/L, m_A_/m_IS_ = 0.2867, A_A_/A_IS_—0.0294.

### 2.2. Development of SIDAs for Hydroxythujones by LC-MS/MS

**Figure 2:**
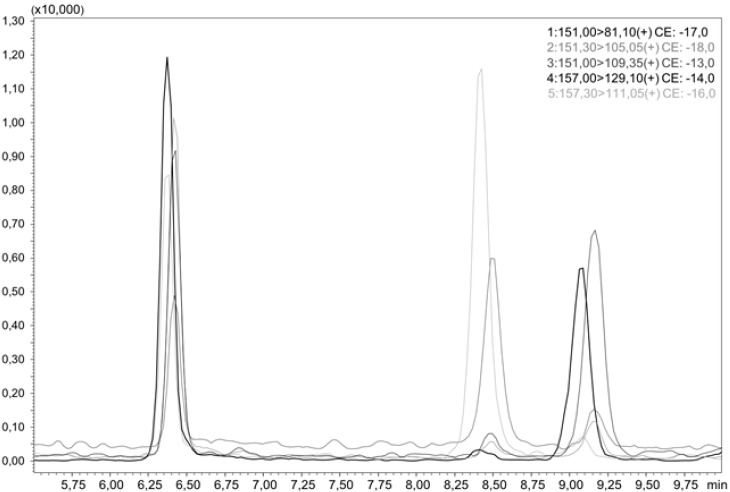
LC-MS/MS chromatogram of an aqueous sample containing 2-OH, *d*_6_-2-OH, 4-OH, *d*_6_-4-OH, 7-OH, and *d*_6_-7-OH.

For the quantitation of the hydroxythujones, liquid chromatographic separation of all analytes is necessary, as they do not differ much in their MS/MS spectra. This was achieved by a smooth gradient of increasing acetonitrile (ACN) in water, as shown in Figure 2, which presents an LC-MS/MS chromatogram of all three hydroxythujones and the respective isotopically labeled isotopologues. To convert the area ratios A_A_/A_IS_ of the analytes and the respective standards to mass ratios m_A_/m_IS_, we analyzed different mixtures of the hydroxythujones with their respective ISs in aqueous solutions, and constructed response curves. The following linear equations with concentration areas were obtained: for 2-OH m_A_/m_IS_ = 0.4136, A_A_/A_IS_—0.005 (12.0–1202.9 µg/L); for 4-OH m_A_/m_IS_ = 0.5772, A_A_/A_IS_—0.003 (3.1–214.2 µg/L); and for 7-OH m_A_/m_IS_ = 0.1753, A_A_/A_IS_—0.007 (3.5–352.0 µg/L).

For the hydroxythujones, conjugation with glucuronic acid or sulfuric acid can be assumed in the human metabolism. By applying enzymatic incubation with a mixture of β-glucuronidase and sulfatase, the hydroxythujones were liberated from these conjugates. The amount of enzymatic solution chosen was similar to that reported by Zeller et al. [5] and Horst et al. [6]. To analyze the hydroxythujones in urine after deglucuronidation, a clean-up procedure was developed using for solid phase extraction (SPE) ENVI-18 cartridges and elution with ACN/water.

### 2.3. Validation

#### 2.3.1. Precision

Repeatability (intra-day precision) and reproducibility (inter-day precision) was investigated by analyzing one human urine sample in triplicate over two weeks. The coefficients of variation for intra-day precision (n = 5) ranged from 2.4% for α-thujone to 12.7% for 4-OH, and for inter-day precision from 3.3% for α-thujone to 16.7% for 4-OH. Due to a lack of human samples containing 2-OH, the determination of repeatability and reproducibility for 2-OH was not possible.

#### 2.3.2. Recovery

A urine sample was spiked with the three hydroxythujones in two different concentration ranges. The recovery was determined after different steps of the sample cleanup. For the evaluation of possible sources of loss during the whole sample preparation, sample workup was conducted and the labeled standards were added at different steps: (0) before workup, (I) after glucuronidase treatment, (II) after protein precipitation and centrifugation, and (III) after SPE cleanup. The results for all steps were compared by setting the results of assay (0) to 100% recovery. The results for both concentration ranges are presented in Table 1, and revealed some variations for the recovery rate of samples, with higher variations at lower concentrations. However, the recovery ranged from 72% to 109%, and confirmed that the absolute losses were negligible. Moreover, even these small losses were compensated for by adding the internal standards at the beginning of the analysis.

#### 2.3.3. Limit of Detection and Limit of Quantification (LOD and LOQ)

The procedure proposed by Vogelgesang and Hädrich [7] was applied to determine the LOD and LOQ. A blank urine sample was spiked with increasing amounts of analytes and IS prior to analysis. The LODs in urine for 2-OH, 4-OH, and 7-OH were 0.98, 0.39, and 0.59 µg/L, and the LOQs were 4.15, 1.28, and 1.82 µg/L. For α-thujone, the LOD (4.7 ng/L) and LOQ (15.6 ng/L) in urine were decisively lower because of the more sensitive detection by SPME-GC-MS.

### 2.4. Human Study

The aim of the present study was to investigate α-thujone metabolism in humans after consumption of a sage tea. A sage infusion was prepared containing 0.64 mg/L α-thujone, which was equivalent to a low extraction rate of almost 6% from the leaves, although falling into the usual extraction range for monoterpenes from 4.8% to 38% [8], and the test persons drank 900 mL. The individual doses for our volunteers were in the range 4.6–14.3 µg/kg bw, and thus were at a factor of almost ten below the recently deduced acceptable daily intake of 110 µg/kg bw/d [9]. After the consumption of the infusion, all volunteers collected their urine in defined time periods.

**Figure 3:**
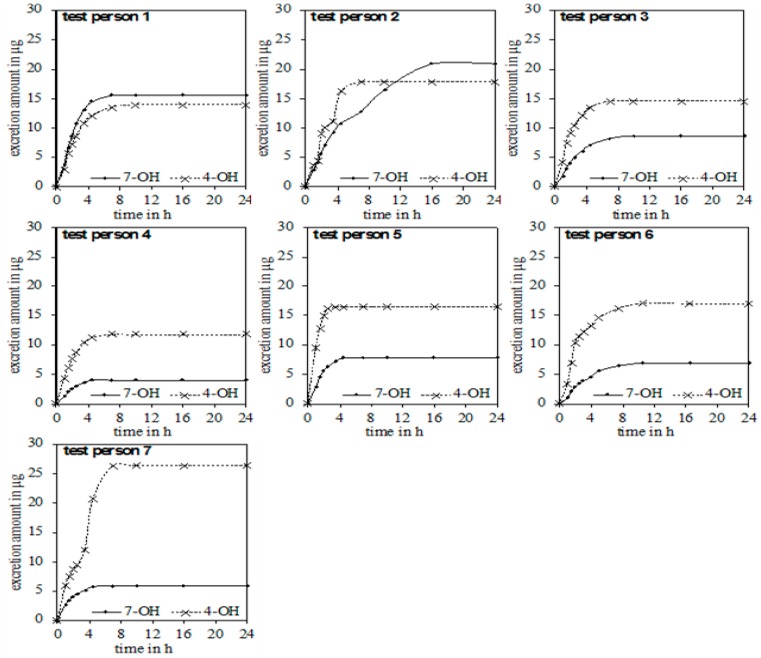
Chronological sequence of the accumulated excretion amount of 4-OH and 7-OH for the seven test persons.

**Figure 4:**
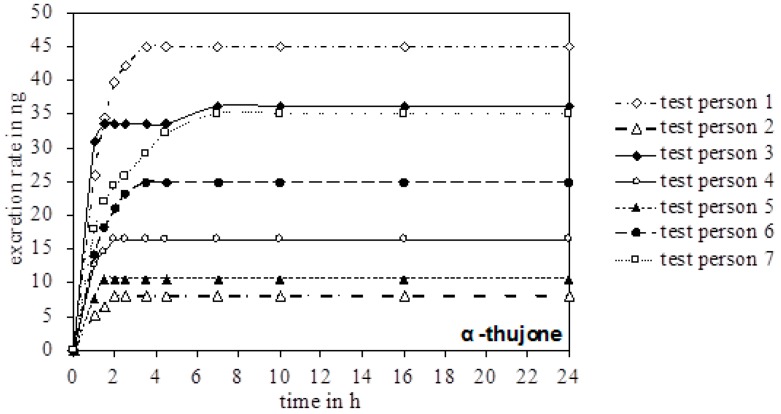
Chronological excretion amount of α-thujone in the urine of all test persons.

In the urine of all test persons, 4-OH and 7-OH, along with α-thujone, were detectable. In none of the samples was the metabolite 2-OH detected. Figure 3 gives an overview of the excreted metabolites of α-thujone under study. In the first hours after ingestion, a fast excretion of 4-OH and 7-OH took place. After 7–8 h no additional excretion was observed. Only test person 2 had a significant slower excretion of 7-OH until 16 h. The parent compound α-thujone showed a low concentration of maximally 94.3 ng/L. Compared to the metabolites, the excretion of α-thujone proceeded faster, thus indicating that its metabolism occurs very effectively. Thus, the concentration of α-thujone after 4.5 h for most volunteers fell under the LODs (Figure 4).

Investigations of phase II metabolism, regarding the α-thujone degree of glucuronidation, showed that 4-OH and 7-OH were found in their free form in urine. For two of the analyzed samples, the concentration of the two hydroxythujones was above the LOQ. From these two samples, a minimum degree of glucuronidation/sulfation for 4-OH (66%) and 7-OH (55%) was calculated.

## 3. Discussion

The study presented here was the first to quantify and balance known metabolites of α-thujone in human urine after administration of an herbal infusion containing the parent compound. α-Thujone and its hydroxylated metabolites were found to be excreted quickly (within 8 h) by almost all volunteers. When summing up the urinary excretion of α-thujone and its hydroxylated metabolites over 24 h, the amount was in the range 15.8–38.8 µg. This means that depending on body weight, only 3.3–7.1% of the α-thujone dose was recovered. The total excreted amounts are given in Table 2, as well as the excretion/bw and dose/bw. From these values, the slight tendency that higher doses lead to higher excretion rates can be concluded. Regarding the distribution of excreted hydroxylated thujones, for almost all volunteers 4-OH was the major isomer, followed by 7-OH. This is in good agreement with the results of Abass et al., who also found that these compounds were the major ones in a low dose range [10]. Only in higher doses, with unmetabolised α-thujone still being present, did the authors in [10] detect 2-OH. This is in good agreement with our finding of 2-OH not being detectable in humans at the low doses applied.

Our results for phase II metabolites showed that 4-OH and 7-OH were found in urine as glucuronides or sulfates, along with their free form. In contrast to this, Abass et al. could not detect these metabolites in human hepatic homogenates. Instead, they detected glutathione and cysteine conjugates without providing any quantitative data [10]. By contrast, Höld et al. determined in mouse urine a degree of glucuronidation of 85% for 2-OH, 61% for 4-OH, and 2% for 7-OH [3].

Obviously, the recovery of the α-thujone dose as its hydroxylated metabolites was as low as 7% and, therefore, decisively lower than the 1,8-cineole (52.5%) recovered as the sum of 2-hydroxy-1,8-cineole, its 9-hydroxy isomer, the 3-hydroxy isomer, and the 7-hydroxy isomer determined by Horst et al. [6]. The fate of the remaining part of the dose is unclear, but in general the difference can be attributed to lower absorption or to still unknown metabolites. As the former is unlikely, due to the rather lipophilic character of thujone (LogP 2.04 [11]), the latter has to be assumed. In this regard, the generation of glutathione or cysteine adducts and formation of mercapturic acids has been hypothesized, but has not been quantitated due to the lack of reference compounds [10].

## 4. Conclusions

With the present study, the chronological excretion of α-thujone, 4-OH, and 7-OH after the consumption of a sage infusion in humans is reported for the first time. Generally, a fast metabolism has the advantage that a potentially toxic substance stays only for a short period in the body. The small amount of detected α-thujone, and the fast excretion of the less toxic hydroxythujones, showed the effective detoxification in humans. It was shown that despite many similarities between the seven test persons, there were individual differences in human metabolism. In general, it is not possible to state one hydroxythujone as the main metabolite in humans. Nevertheless, 2-OH apparently is negligible in human metabolism of α-thujone.

## 5. Material and Methods

### 5.1. Chemicals

α-Thujone, the hydroxythujones 2-OH, 4-OH and 7-OH and their isotopically labeled compounds were prepared according to Thamm et al. [4]. Acetonitril (LC-MS grade) and water (LC-MS grade) were purchased from VWR (Radnor, PA, USA). β-Glucuronidase (type HP-2 from Helix pomatia) and hydrochloric acid (conc.) were purchased from Sigma Aldrich (St. Louis, MO, USA). Sodium chloride was purchased from Merck KGaA (Darmstadt, Germany).

### 5.2. Sage and Sage Infusion

Sage (*Salvia officinalis* L.) was provided by the German Research Center for Food Chemistry and purchased from the Mediflor GmbH (Landshut, Deutschland). It was seeded in Italy and grown in a greenhouse (at least 25 °C, 8 h sun). The freshly harvested sage was dried at 50 °C for 24 h and chopped in a mixer (DPA141 Moulinex, Frankfurt/M., Germany). The homogenized sage was sealed in an airtight pack, and stored in the dark until preparation of the sage infusion. The α-thujone content (1604 mg/kg) of the sage was analyzed by Jonas et al. [12]. To prepare the infusion, the dried sage was weighed (6.00 g) into a teapot and brewed with 900 mL of boiling water. After letting it steep for 5 min, the infusion was filtered.

### 5.3. Design of the Human Study

The protocol of the study was approved by the Ethics Committee of the Faculty of Medicine of the Technische Universität München (1996/07). Written informed consent was obtained from participants before inclusion into the study. Seven healthy, non-smoking volunteers, six Caucasian and one Asian, participated in the study (3 men and 4 women, mean (±standard deviation [SD]) age 29.7 (±9.21) years, and mean (±SD) body mass index 23.1 (±5.08) kg/m^2^). For 24 h prior to the study, the volunteers avoided herbs and spices containing α-thujone for a wash out. A blank urine sample was collected as a control from each volunteer before consumption of the sage infusion. On an empty stomach, the volunteers drank the tea (575 µg α-thujone) within 10 min, and urine was collected at 1, 1.5, 2.5, 3.5 h, and in the time frames of 4.5–6, 6–8, 8–14, 14–24 h after consumption. A defined meal (cheese sandwich) was eaten after 2 h. The samples were stored at 4 °C until preparation for analysis.

### 5.4. Analysis of Hydroxythujones in Urine

#### 5.4.1. Urine Sample Preparation

The cooled samples were adjusted with HCl to pH 5 and divided into 2 mL aliquots. A solution containing the labeled hydroxythujones (220–430 ng) and β-glucuronidase (5000 units/mL sample; 1 unit liberates 1.0 mg phenolphthalein from its glucuronide per hour at pH 5.0 at 37 °C) was added, and the mixture stirred at 37 °C for 15 h. Afterwards, the samples were centrifuged (16000 rpm, 4 °C, 15 min) and the supernatant was subjected to solid phase extraction (SPE).

#### 5.4.2. SPE

The SPE tubes (ENVI-18, 100 mg, Supelco) were equilibrated with 2 × 1 mL methanol and 2 × 1 mL water. Thereafter, the clear supernatant was loaded on the columns and was slowly passed through without suction. The protein residue was reextracted with water/methanol (95:5, 1 mL), and also loaded on the SPE tubes. The tubes were washed with water/methanol (95:5, 2 × 1 mL) by suction, and then dried carefully. Analytes were slowly eluted with water/acetonitrile (50:50, 500 µL). The extracts were analyzed by LC-MS/MS.

#### 5.4.3. LC-MS/MS

Measurements on the LC-MS/MS were performed using a Shimadzu LC-30A Prominence system (Shimadzu, Kyoto, Japan) interfaced to an LCMS-8050 Triple Quadrupol mass spectrometer (Shimadzu, Kyoto, Japan). A total of 40 µL was injected onto a YMC Pro Pack C4 at 30 °C oven temperature and flow of 0.5 mL/min with water (solvent A) and ACN (solvent B). The gradient was started and held at 5% B for 2 min, raised linearly from 5% B to 30% B during the next 1 min, then raised linearly to 33% within the next 7 min, and to 100% B within the next 1 min. Thereafter, the mobile phase returned to 5% B within 2 min, and the system was equilibrated for 18 min before the next run. The atmospheric pressure chemical ionization (APCI) source was operated in the positive mode under the following conditions: nebulizing gas flow 3 L/min; interface temperature 350 °C; desolvation line temperature 200 °C; drying gas flow 5 L/min. For MS/MS of the hydroxythujones, the mass transitions (*m/z* precursor ion/ *m/z* product ion) 151/81 (collision energy = −17 V) and 151/123 (−14 V) for 2-OH, 151/109 (−13 V) and 151/91 (−23 V) for 4-OH, 151/105 (−18 V) and 151/81 (−17 V) for 7-OH, 157/129 (−14 V) and 157/87 (−15 V) for *d*_6_-2-OH, 157/11 (−16 V) and 157/91 (−23 V) for *d*_6_-4-OH, and 157/129 (−14 V) and 157/99 (−16 V) for *d*_6_-7-OH were used.

#### 5.4.4. Calibration and Calculation

To determine the hydroxythujone content in urine, calibration functions for 2-OH, 4-OH, and 7-OH were generated with the respective labeled compounds. Calibrators with unlabeled and labeled compounds were prepared in an aqueous solution, in seven different ratios for the unlabeled compounds ranging from 3.1–601.9 µg/mL, while maintaining the labelled compounds constant at concentrations of 450 µg/mL each. The samples were measured by LC-MS/MS. From the relation of area ratios to molar ratios, calibration curves were constructed. The corresponding linear equation was checked for linearity (adjustment by Mandel), and used to calculate the concentration of the analytes in samples by considering the area ratio, added amount of labeled standard, and the sample volume.

#### 5.4.5. Precision

Intra- and inter-assay precision was checked by quintuplicate determination of the hydroxythujone content of a urine sample on three different days within 1 week.

#### 5.4.6. Stability

Stability of the labeled compounds during workup was verified by adding labeled and unlabeled compounds to a blank urine sample. Aliquots were stored at pH 5.0 for 1 d, 2 d, or 3 d at 4 °C. The fresh urine sample was also analyzed directly, and the resulting area ratio of the analytes to standards was compared with those of the stored samples.

#### 5.4.7. LOD and LOQ

Urine, which was devoid of the analytes under study, was used for determination of the LOD and the LOQ. The following amounts of analytes were added to the respective matrices: 1.7–12.0 µg/L 2-OH: 0.5–3.1 µg/L 4-OH und 0.5–3.5 µg/L 7-OH. Each sample was analyzed in triplicate by SIDA as described above. LOD and LOQ were determined according to the method of Vogelgesang [13]. LOD is the addition value referring to the upper 95% confidence limit of the calibration line at the zero addition level. LOQ is the addition level of the lower 95% confidence limit, which meets the upper 95% confidence limit of the addition level at LOD.

#### 5.4.8. Recoveries of Analytes during Workup

To a blank urine sample two different amounts of the analytes 2-OH, 4-OH, and 7-OH were added and analyzed with four different assays (each in duplicate), which were varied when the internal standards were added. The internal standards were either added at the beginning of sample workup (0), after the incubation time for glucuronide hydrolysis (I), after the protein precipitation (II), and after the SPE directly before LC-MS/MS analysis (III). The results were compared by setting assay (0) to 100% recovery.

### 5.5. Analysis of α-Thujone in Urine

#### 5.5.1. Urine Sample Preparation

The urine samples (5 mL) were added to 2.00 g NaCl in 20 mL headspace vials. A total of 100 µL of the internal standard was added, and the vials were sealed with a septum crimp cap before being equilibrated for at least 60 min prior to analysis.

#### 5.5.2. SPME-GC-MS

Analysis was performed using an Agilent 7890B GC (Agilent Technologies, Santa Clara, CA, USA) coupled with an ion trap mass spectrometer Agilent 240 (Agilent Technologies, Santa Clara, CA, USA), with methanol as the chemical ionization gas and an ionization energy of 70 eV. For solid phase microextraction a Car/PDMS 75 µm fiber (Supelco, Bellafonte, PA, US) was used. The fiber was exposed to the sample headspace for 30 min at 70 °C, and then desorbed at injector at 310 °C for 5 min. The fiber was then conditioned in the injector at 300 °C, with a split of 40 mL/min for 30 min. A DB column (VF-5MS, 25 m × 250 µm × 0.25 µm) was used with the following oven temperature program: 40 °C for 2 min, 6 °C/min to 90 °C for 5 min, 3 °C/min to 140 °C, 30 °C/min to 240 °C. For quantification, the following mass traces were used: α-thujone (*m/z* 135) and *d*_6_-α-thujone (*m/z* 141).

#### 5.5.3. Calibration and Calculation

For calibration, 5 mL aqueous solutions were prepared in 20 mL headspace vials with 2 g NaCl in eight different concentrations of the unlabeled α-thujone, ranging from 0.01–0.67 µg/mL, and a constant concentration of the labeled compound (6 ng). The solutions were analyzed as described above. From the relation of area ratios to molar ratios a calibration curve was constructed, and the corresponding linear equation was checked for linearity (DIN 38402, part 51—adaption of Mandel and residual analyzes) and used to calculate the concentration of the analytes in samples by considering the area ratios, added amount of labeled standard, and the sample weights.

#### 5.5.4. Precision

Intra- and inter-assay precision was checked by quintuplicate determination of the Hydroxythujone content of a urine sample on two different days within 1 week.

#### 5.5.5. Stability

The stability of the labeled compounds during analysis was verified by repeating the calibration analysis, with the solution in two different concentrations being stored at 70 °C for 1 day, and at room temperature for 5 days in the dark before GC-MS analysis. The resulting area ratio of the analyte to standard was compared with that of the mixture measured directly after preparation.

#### 5.5.6. Determination of Detection and Quantification Limits

Detection and quantification limits were determined according to Vogelgesang and Hädrich [7]. The analyte α-thujone was added to a blank urine sample and 2.00 g NaCl in four different concentrations, ranging from 2.7–27.1 ng/L, in quadruplicate, and analyzed as described above.

## Figures and Tables

**Table 1:** Absolute recoveries of the hydroxythujones after different steps of the workup: (I) after glucuronidase treatment, (II) after protein precipitation and centrifugation, and (III) after SPE cleanup.

Concentration Range (µg/L)	93.3–173.4			17.8–49.2		
Recovery (%)	After Step			After Step		
	I	II	III	I	II	III
7-OH	97	95	91	80	80	78
4-OH	94	92	91	106	98	100
2-OH	93	91	88	106	109	72

The concentration determined by stable isotope dilution assays (i.e., by adding the internal standards before workup) was set to 100%.

**Table 2:** Total excretion of α-thujone and the hydroxymetabolites in human urine after the consumption of sage infusion.

Test Person	1	2	3	4	5	6	7
4-OH (µg)	13.9	17.9	14.6	11.8	16.5	17.0	26.3
7-OH (µg)	15.6	20.9	8.6	4.0	7.8	6.9	5.9
α-Thujone (ng)	45.1	8.0	36.1	16.3	10.7	25.0	35.1
excretion/bw (µg/kg)	0.6	0.7	0.3	0.2	0.3	0.2	0.5
dose/bw (µg/kg)	14.3	11.4	8.2	6.7	8.9	4.6	7.6
